# Endoparasites of *Leopardus pardalis* (Carnivora, Felidae) rescued in the State of Rio de Janeiro

**DOI:** 10.29374/2527-2179.bjvm006123

**Published:** 2023-12-20

**Authors:** Gabriela Pereira Salça de Almeida, So Yin Nak, Gabriel Alcides Capucho, Brena Gava Guimarães, Diefrey Ribeiro Campos, Debora Azevedo Borges, Daniel de Almeida Balthazar, Thais Ribeiro Correia

**Affiliations:** 1 Veterinarian, MSc. Programa de Pós-Graduação em Ciências Veterinárias (PPGCV), Departamento de Parasitologia Animal (DPA), Instituto de Veterinária (IV), Universidade Federal Rural do Rio de Janeiro (UFRRJ). Seropédica, RJ, Brazil.; 2 Veterinarian, MSc. Autonomus, Manus, AM, Brazil.; 3 Veterinarian, MSc. Autonomus, Curitiba, PR, Brazil.; 4 Veterinarian, DSc. PPGCV, DPA, IV, UFRRJ. Seropédica, RJ. Brazil; 5 Veterinarian, DSc. Departamento de Medicina e Cirurgia Veterinária (DMCV), IV, UFRRJ. Seropédica, RJ, Brazil.; 6 Veterinarian, DSc. DPA, IV, UFRRJ. Seropédica, RJ, Brazil.

**Keywords:** ocelot, diagnosis, endoparasitosis, coproparasitological, jaguatirica, diagnóstico, endoparasitoses, coproparasitológico

## Abstract

Wild cats play an important role as top predators in the food chain and act as ecosystem regulators. However, in recent decades, many studies have demonstrated the potential effects of parasitic diseases on wild carnivore populations, including cats. This study reports on the endoparasites found in an injured and rescued specimen of *Leopardus pardalis* in the state of Rio de Janeiro. Fecal samples were collected and processed using five coproparasitological techniques: a simple flotation centrifugation, a zinc sulfate flotation centrifugation, a formalin-ether sedimentation centrifugation, a conical centrifuge tube technique, and a modified Ziehl-Neelsen staining technique for fecal smears. Helminth eggs belonging to the families Trichuridae and Diphyllobothriidae and the genus *Toxocara* were found in both sedimentation flotation techniques. Protozoan oocysts belonging to the genus *Cryptosporidium* were identified by modified Ziehl-Neelsen staining. These findings show that ocelots can harbor potentially zoonotic and pathogenic endoparasites. Further studies on the helminths and protofauna of these animals are necessary.

## Resumo

Os felinos selvagens desempenham um papel importante como predadores de topo na cadeia alimentar e atuam como reguladores do ecossistema. No entanto, nas últimas décadas, muitos estudos demonstraram os efeitos potenciais de doenças parasitárias em populações de carnívoros selvagens, incluindo gatos. Este estudo relata os endoparasitos encontrados em um espécime ferido e resgatado de *Leopardus pardalis* no estado do Rio de Janeiro. Amostras fecais foram coletadas e processadas usando cinco técnicas coproparasitológicas: uma centrifugação por flotação simples, uma centrifugação por flotação com sulfato de zinco, uma centrifugação por sedimentação com éter de formalina, uma técnica de tubo de centrífuga cônica e uma técnica modificada de coloração de Ziehl-Neelsen para esfregaços fecais. Ovos de helmintos pertencentes às famílias Trichuridae e Diphyllobothriidae e ao gênero *Toxocara* foram encontrados em ambas as técnicas de flotação por sedimentação. Oocistos de protozoários pertencentes ao gênero *Cryptosporidium* foram identificados pela coloração de Ziehl-Neelsen modificada. Esses achados mostram que jaguatiricas podem abrigar endoparasitas potencialmente zoonóticos e patogênicos. Mais estudos sobre os helmintos e a protofauna desses animais são necessários.

## Introduction

As top predators, wild cats are important to the fauna and act as regulators of the ecosystem. The medium-sized felid *Leopardus pardalis*, popularly known as the ocelot, is widely distributed between the southern United States and northeastern Argentina (Wang et al., 2019). It is listed as “endangered” in Appendix I of the International Convention on International Trade in Species of Wild Flora and Fauna (Convenção Internacional Sobre Comércio Internacional das Espécies da Flora e Fauna Selvagem em Perigo de Extinção, 2016) and considered in the category of “least concern” by the União Internacional para a Conservação da Natureza e dos Recursos Naturais (2016). This carnivore species was often considered abundant, reaching high population densities with 94.7 ocelots/100 km^2^ in a protected area (Kolowski & Alonso, 2010), and can survive in anthropized environments (Di Bitetti et al., 2008). However, the decline of natural habitats, hunting, and roadkill incidents have drastically reduced their numbers (Oliveira et al., 2013).

Parasitism is a threat for wild carnivore populations because can control animal population dynamics. In the same way as a predator, parasites can affect an abundance of different animals’ species, considering the morbidity and mortality that parasitic diseases can cause in situations of imbalance (Thompson et al., 2010).

Parasitism does not always characterize a disease, however, there are cases in which this relationship causes damage to the hosts. Damage may lead to metabolic deficits usually caused by the high parasite loads or by parasites that trigger diseases with which the host's immune system may be naïve (Neves, 2003). This justifies the inclusion of parasitism as a biotic force capable of determining the biodiversity of communities, providing new structural profiles (Poulin, 1999; Poulin & Morand, 2004). Thus, parasitic diseases can directly interfere with the felid community and other populations. Depending on the parasite species, there may be a reduction in host feeding activity, animal weakness, and mortality. Consequently, studies on intestinal parasites of wild felids contribute to the knowledge of felid-biology and the associations of felids with their environment. Additionally, they can provide information on the quality of life of wild cats as parasitic diseases greatly interfere with these animals physically and behaviorally (Poulin & Morand, 2004).

This study investigated the presence of parasitic forms during different parasitological examinations of fecal samples of a *L. pardalis* treated at the Veterinary Hospital of the Universidade Federal Rural do Rio de Janeiro.

## Material and methods

In March 2018, a young debilitated male wild *L. pardalis* was found on a farm located in the state of Rio de Janeiro and sent to the Wild Animal Triage Center (CETAS) in the municipality of Seropédica. The animal was in a semi-comatose state with spikes in its oral cavity and myiasis in the palate region. The spikes were possibly acquired by hunting *Coendou prehensilis*, popularly known as porcupines. Emergency care was given, the wound cleaned, and the animal was brought to an enclosure for recovery. The animal could not be reintroduced to its natural habitat due to the loss of its soft palate, a nasopharyngeal stenosis resulting from the scarring process, and a chronic sinusitis.

Fecal samples were collected daily from the enclosure soon after natural defecation and sent within 24 hours to the **“hidden for review”** for coproparasitological tests.

The forwarded fecal samples were homogenized and processed using five coproparasitological techniques per sample: (1) the simple centrifugation-flotation method; (2) the centrifugation-flotation technique in zinc sulfate, which concentrates floating nematode eggs and cysts and protozoa oocysts; (3) the formalin-ether concentration technique, which is based on centrifugation-sedimentation to investigate the presence of trematode eggs, cestoides, and nematodes (Monteiro, 2011); (4) the cone tube centrifugation technique, specific for lung larvae research (Ueno & Gonçalves, 1998); (5) a fecal smear was examined for *Cryptosporidium* spp. oocysts using a modified Ziehl-Neelsen staining technique (Luna, 1968). All slides were analyzed under an Olympus BX51 microscope with an UC30 camera at magnifications ranging from 10x to 100x and evaluated using CellSens Dimension software. Thereafter, visual examination was performed to identify the parasites based on their structures, specifically, their egg morphologies. All techniques used to extract and identify the parasitic forms were in accordance with Ueno and Gonçalves (1998) and Sloss et al. (1999).

## Results

**Figure 1:**
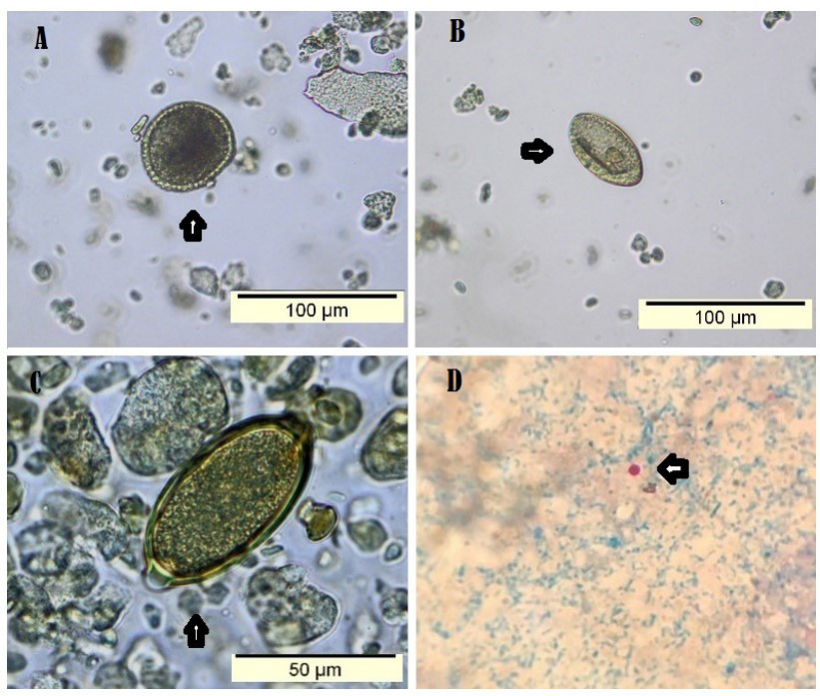
*Toxocara* spp. eggs (A), eggs with morphology consistent with eggs from the Diphyllobothriidae family (B), eggs with morphology consistent with eggs from the Trichuridae family (C), and a *Cryptosporidium* spp. oocysts (D). All found in a *Leopardus pardalis* fecal smear stained with a modified Ziehl-Neelsen technique.

During the coproparasitological analysis, three helminth morphospecies of epidemiological importance were found (Figure 1): nematode eggs of the genus *Toxocara*, eggs with a morphology consistent with the Trichuridae family, and eggs with a morphology consistent with the Diphyllobothriidae family. Additionally, *Cryptosporidium* spp. oocysts were identified in the stool samples stained using a modified Ziehl-Neelsen technique (Elsafi et al., 2014; Tahvildar-Biderouni & Salehi, 2014).

To identify the parasite species, six eggs of each genus or family were measured as described in Table 1. Measurements were taken up to 24 hours after the coproparasitological techniques were completed. Morphological identification was performed as described by Soulsby (1982).

**Table 1:** Morphometry of helmith eggs found in a *Leopardus pardalis* feces. Rio de Janeiro, Brazil.

Eggs	Diphyllobothriidae		*Toxocara* sp.		Trichuridae	
	width (µm)	Length (µm)	width (µm)	length (µm)	Width (µm)	length (µm)
Egg 1	35.86	55.77	59.71	78.98	34.04	78.79
Egg 2	36.38	56.17	69.00	77.76	33.81	76.93
Egg 3	34.08	59.06	59.17	67.90	35.43	74.60
Egg 4	36.01	58.55	62.43	70.37	35.60	71.34
Egg 5	40.87	57.93	56.68	58.60	33.95	77.49
Egg 6	32.75	56.29	56.95	58.60	26.29	52.85
**Average** 1	**35.99**	**57.30**	**60.66**	**68.70**	**33.19**	**72.00**
**Standard deviation**	**2.76**	**1.39**	**4.59**	**8.89**	**3.47**	**9.74**

1 arithmetic average.

The eggs belonging to the family Diphyllobothriidae ranged from 32.75 to 40.87 µm in width and 55.77 to 59.06 µm in length, whereas the eggs belonging to the family Trichuridae ranged from 26.29 to 35.60 µm in width and 52.85 to 78.79 µm in length. The eggs belonging to the genus *Toxocara* sp. ranged from 56.68 to 69.00 µm in width and 58.60 to 78.98 µm in length.

The investigation of larvae in the coproparasitological examination of *L. pardalis* stool samples using the conical centrifuge tube technique yielded negative results.

## Discussion

Morphological details and the range and length and width distributions are key aspects in the identification of eggs and larvae using light microscopy. Minor details can be used to identify distinct taxonomic levels; however, morphometric similarities and overlaps often make accurate identification difficult. Coproparasitological examinations have limitations. It is not always possible to identify a species because of the insufficient records in the literature describing the morphology of eggs/cysts or oocysts. However, coproparasitological diagnostic techniques are important tools that enable the evaluation of the intensity of infections, which allows the probability of transmission between individuals in the same community to be verified, or to evaluate the sanitary conditions of the populations and the impact of control measures (Mascie-Taylor et al., 1999).

In this study, we identified operculated eggs, whose appearance was consistent with that of cestoids belonging to the family Diphyllobothriidae. Morphological analysis of the corresponding adult specimens was not possible, making it difficult to differentiate the detected eggs from eggs of the genera Spirometra and *Diphyllobothrium*. The eggs found in this study can easily be confused with eggs of trematode specimens of the genus *Alaria*. However, by measuring the eggs we concluded that they were indeed cestoids belonging to the family Diphyllobothriidae parasitizing *L. pardalis.* The eggs were half the size of an average egg of the genus *Alaria* (100 µm long and 56 µm wide) and had the same egg morphology as an egg found in the feces of *L. weidii* described by Marques et al. (2019).

The results from two coproparasitological techniques, the centrifugation-sedimentation in formalin-ether and the simple centrifugation-flotation, were positive: both with the presence of eggs, dark brown in color, ovoid, not embryonated, and with an operculum visible at one end suggestive of *Spirometra* spp. The average size of the six eggs was 57.30 μm long and 35.99 μm wide. Eggs can be identified in feces 12 days after infection of the host, with the release of approximately 70,000 eggs per gram of feces (Vieira et al., 2008).

McGlade et al. (2003) reported that eggs belonging to the family Diphyllobothriidae often have an underestimated prevalence in animals because of the low sensitivity of the diagnostic methods used in routine laboratory work. However, this statement is controversial, because unlike eggs of the classes Cestoda and Trematoda, which can only be visualized using the sedimentation method, the flotation method, employing a saturated sugar solution with a specific density of 1.280, allows for an accurate diagnosis, as shown in this study. Perhaps the high parasite loads in the samples allowed for this finding. Most likely, felids can extend periods of negativity, followed by periods of positivity (Marques et al., 2019). Therefore, periodic fecal examinations should be performed on animals in at-risk areas or those with predisposing factors.

Eggs of Trichuridae specimens were identified using the formalin-ether centrifugation-sedimentation, simple centrifugation-flotation, and zinc sulfate centrifugation-flotation techniques. The average egg size was 72 × 33 μm. These eggs were bioperforated and barrel-shaped with lateral symmetry, suggestive of *Trichuris* spp. The similarities in egg shapes of different species or genera, such as *Trichuris* and *Capillaria*, can make a differentiation difficult. Variations in egg sizes of the genus *Trichuris* was observed in one study, where smaller eggs of approximately 57 × 26 μm and larger eggs reaching 78 × 30 μm were observed (Yoshikawa et al., 1989). Accurate identification and parasitological diagnosis is limited by such variations and affected by the technique itself, as different solutions used for different techniques can generate distortions in egg size.

*Toxocara* spp. eggs found in the feces of *L. pardalis* averaged 60.66 μm wide and 68.70 μm long, compatible in size with those previously reported parasitizing other wild felid species such as *L. colocolo*, *L. geoffroyo*, *L. tigrinus*, *Pu. yagouaroundi* (Gallas & Silveira, 2011), *Pa. onca*, *Pu. concolor*, and *L. pardalis* (Vieira et al., 2008). These authors were able to differentiate between *T. cati* and *T. canis* species by detection of adult specimen in the feline feces and through parasitological necropsy techniques performed on felid carcasses. However, this report corroborates the findings of Bally et al. (2021) using only fecal samples for diagnosis.

Although *L. pardalis* presented with respiratory symptoms that could be related to the lesions on the palate, the respiratory signs stemming from a parasite infection cannot be ruled out once sample collection time may have prevented the detection of larvae.

Some studies have shown that domestic cats are hosts of *Cryptosporidium* spp. (Huber et al., 2002) however, we did not find reports on this protozoan parasitizing *L. pardalis.* Moreover, there were no reports on the use of the modified Ziehl-Neelsen technique as a routine detection method for *Cryptosporidium* spp., which may suggest an absence of findings when this technique is not used, possibly indicating the inadequacy of the choice of technique for *Cryptosporidium* spp. diagnosis.

The diverse diet of *L. pardalis*, which includes small mammals, birds, reptiles, and insects (Ludlow & Sunquist, 1987; Moreno et al., 2006; Oliveira et al., 2010) may contribute to infection by several species of parasites. Therefore fecal samples of *L. pardalis* should be further studied and perhaps included as environment quality sentinel once more than 45% of zoonotic pathogens include a carnivorous host in their life cycle.

Generally, the helminthofauna and protofauna of wild cats are poorly understood. Much of the literature on the parasitology of wild cats stems from animals in captivity or adult parasites found in carcasses through necropsy. Therefore, it is essential to monitore the health status of these animals and their surrounding parasitological fauna through partnerships with wildlife screening centers (such as CETAS) and veterinary hospitals that serve wildlife.

## Conclusions

These findings show that ocelots potentially play an important role in the maintenance and transmission of endoparasites in the wild, domestic and even human animals, as well as the reverse. Therefore, further studies and analyzes of the biodiversity of helminths and the protofauna of these animals are necessary.
